# Predicting Scholars' Scientific Impact

**DOI:** 10.1371/journal.pone.0049246

**Published:** 2012-11-21

**Authors:** Amin Mazloumian

**Affiliations:** 1 ETH Zürich, Department of Humanities and Social Sciences, Chair of Sociology, in particular of Modeling and Simulation, Zürich, Switzerland; Aalto University, Finland

## Abstract

We tested the underlying assumption that citation counts are reliable predictors of future success, analyzing complete citation data on the careers of 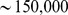 scientists. Our results show that i) among all citation indicators, the annual citations at the time of prediction is the best predictor of future citations, ii) future citations of a scientist's published papers can be predicted accurately (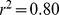 for a 1-year prediction, 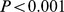) but iii) future citations of future work are hardly predictable.

## Introduction

Many decisions with regard to the allocation of research funds and the assignment of positions are based on citation counts [1]–[4]. Citation counts are considered for awarding post-doctoral fellowships, assigning junior faculty positions and tenures [5]–[10]. However, it remains unclear whether citation-based indicators are appropriate measures to judge a scientist's future research quality [1], [11].

In this study, we analyzed complete panel data on the careers of more than 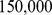 scientists. Considering various metrics of research quality, we tested the assumption that citation counts are reliable predictors of future scientific success, as measured by future citations. Recent studies have partially measured the predictive power of several citation indicators for scientists' future citations [1], [11]–[13]. However, because of the limited availability of data these analyses are performed on a small population of scientists, and hence cannot establish with confidence the connection between past and future citations. “There have been few attempts to discover which of the popular citation measures is best and whether any are statistically reliable” and “existing databases such as the ISI can therefore actively help to improve the situation by compiling field-specific homogeneous data sets similar to what we have generated for SPIRES” [1].

We considered a range of bibliometric indicators to assess scientists' research quality. Productivity and impact are the two main dimensions of research quality [14]–[17]. Some indicators such as the number of published papers and the mean annual number of publications only reflect scientists' productivity. Citation-based indicators, on the other hand, are used to index impact both at the level of single publications [18]–[20] and over individuals' careers (for example a scientist's mean citation per paper, or total number of citations) [5], [21]–[24]. However, the probability of an article being cited depends on various factors (e.g. time, field, journal, availability of the article, authors'social network) [24]–[27].

Hirsch proposed the widely-used *h* index, which combines both productivity and impact [12]. A scientist's *h* index value is defined as the maximum Natural number 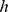 for which the scientist has 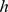 papers with at least 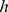 citations. This gives a lower bound of 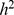 citations to the scientist. In comparison with the cumulative number of citations, the *h* index is not critically inflated by a small number of highly cited papers. In the same study, Hirsch defined the *m* index as a scientist's *h* index value divided by the time (years) elapsed from the first publication of the focal scientist [12].

The applicability of *h* to evaluate scientists has been heavily investigated in the literature [12], [28], [29]. High profile scientists (e.g. Nobel laureates and members National of Academy of Sciences) generally score higher *h* index values. Bornmann and Daniel tested its applicability to junior scientists and showed that the decision of a peer-review committee to award long-term fellowships favored those applicants with higher *h* index values [30].

A similar citation indicator that combines productivity and impact is the *g* index [31]. A scientist's *g* index value it the highest number 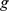 of papers that receives 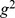 or more citations. By definition for every scientist 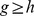. The index inherits some good properties of the *h* index [32], The index has very different value than the *h* index for those who published few highly cited articles.

## Results

**Figure 1:**
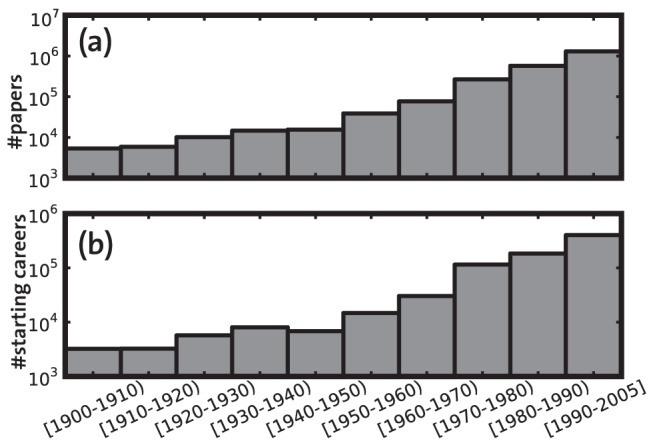
Histograms of a) number of papers per decade and b) number of starting careers per decade. The publication year of a scientist's first paper is considered as the starting year of her career.

We extracted citation information on the careers of 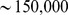 scientists from the Thomson Reuters Web of Science dataset. The careers comprise about 2 million papers and around 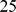 million citations of the papers since 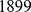. The number of papers per decade and the number of starting careers per decade are shows in Fig. 1. We used publication year, author list and list of references of the papers from the Thomson Reuters Web of Science dataset. Author names appeared as pairs of family name and initials (e.g. “S Genoud”). For some of the more recent journals, full first names of authors were also provided. With our dataset, we therefore faced the name ambiguity problem, i.e. an initial may refer to more than one unique author, and an author may have more than one initial. Name ambiguity is a big hurdle in analyzing individual careers for which there exist no standard solution [33]–[35]. A method applicable to one dataset may not perform well for another.

In our study, instead of solving the complicated name ambiguity problem, we avoided it by discarding author names that appeared with different initials. For instance, because “A Smith” and “B Smith” both exist in our dataset of more than 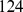 million initials, we discarded family name “Smith”, whereas family name “Ambonati” was selected because only one initial “M” was associated with it. This not only removes frequent family names, but also authors with different initials' spellings (e.g. “A Smith”, “AH Smith”, “HA Smith” may actually refer to the same author).

This procedure resulted in extracting more than one million family names associated with unique initials, for a total of about 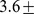 million entries. Nevertheless, a family name with a unique initial may still refer to at least two authors with different first names (e.g. both Marco Ambonati and Mario Ambonati have initial M). By analyzing the papers for which full first names were also provided, we estimated the probability of such cases to be 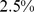. There is also a miniscule probability that a family name with a unique initial and a unique first name belongs to at least two different authors. However, estimating this probability is impossible with our current data. We performed our analysis on more than 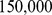 scientists whose career length, calculated as the time gap between the first and the last paper, was longer than 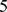 years. Our results were not sensitive to the minimum career-length selection criteria.

**Figure 2:**
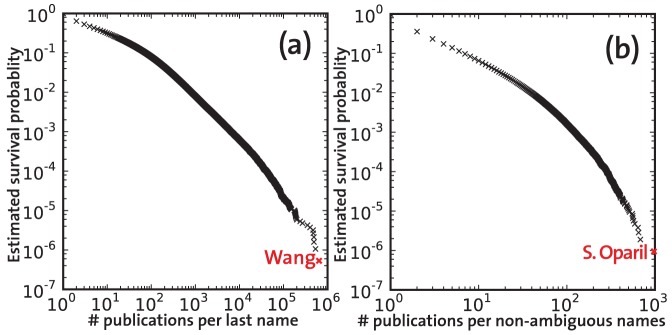
Effect of removing authors with ambiguous names. a) Cumulative distribution of the number of occurrences of family names in the author lists of distinct papers before the removal. b) Cumulative distribution of number of papers per scientists after removing ambiguous names.

The result of ambiguity removal procedure is demonstrated in Fig. 2. The most ambiguous family name (“Wang”) appeared in the author lists of about 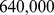 papers, and obviously does not refer to a unique author (Fig. 2a). After the removal of ambiguous names, the maximum frequency of a last name with unique initial is 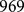 for the name “S Oparil”, as shown in Fig. 2b. Moreover, the general statistics of the selected papers such as the mean number of authors per paper (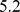) or the mean number of references per papers (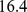) remained the same.

**Figure 3:**
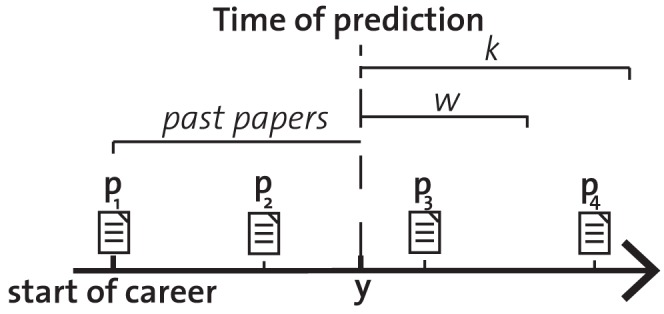
A schematic career for a scientist with 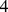
 **papers** 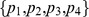 **.** We consider her career from her first paper 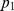. At prediction point 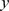, we estimate the citations received in 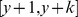 of both past papers (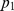 and 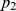), and of future papers published in 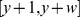 (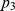). Paper 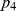 is a future paper which is not published in time-window 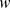, and therefore excluded for the time-windows as defined by 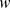 and 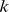.

At every year 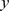 during a scientist's career, our goal is to estimate two quantities: a) the total citations received by her papers published until and including year 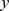, in the 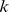 subsequent years 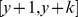, and b) the citations of her papers published in the 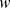 subsequent years 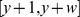, received in the 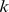 subsequent years 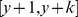. For 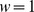 and 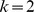, for example, we estimated citations to the papers published in the year 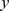 received in the two years 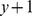 and 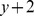. Obviously, the time of prediction 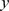 varies between the publication year of her first to last paper (Fig. 3). Papers published before the time of prediction were treated as past papers and papers published afterwards as future papers. Obviously, future citations may refer to both past papers and future papers. Because the information about past citations of past papers is available at the time of prediction, estimating future citations of past papers is easier.

The information that we used in our model is the value of 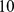 prominent citation indicators at the time of prediction, namely the number of papers, the total number of citations, the career length, the average number of published papers per year, the average annual citations, the annual citations at the time of prediction, the average citations per paper, the *h* index, the *m* index, and the *g* index.

The prediction points were time-lagged according to 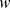 and 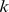. For 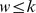, every 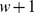 year we added a prediction point. For 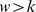 the problem reduces to the case when 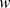 is equal to 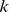. Because no paper published after the 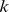-th year receives citations within the first 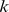 years. The earliest prediction point was 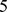 years after the publication year of first paper. We therefore excluded the scholars with careers shorter than 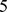 years and the initial years (which may include graduate and PhD studies) of scholars with longer careers. This gave us between 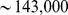 (for 10-year predictions of 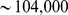 long careers) and 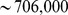 (for 1-year predictions of all careers) prediction points.

For example, suppose a scientist's first paper was published in 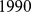 and her last paper was published in 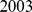. For 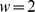, we chose prediction years at 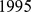, 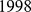, and 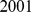. The corresponding future citations periods were then 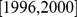, 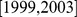, and 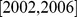. Although consecutive samples overlap in citation period, no citation is counted more than once. Because selected papers do not overlap in consecutive samples.

Due to the nested structure of data (within-person time observations), we used multi-level regression models with random effects at the individual level. We implemented the models in “STATA” software using the “xtreg” function with the “mle” option. All variables were added in log scale.

More specifically, we estimated for scholar 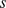 the citations to a certain subset of his papers (selected by time-window 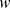) in 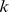 subsequent years using citation indicators 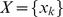 as
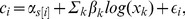
(1)where 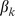 is the coefficient of citation indicator 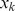 and 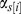 is the intercept estimated for scholar 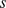. Note that intercepts of this model are independently estimated for individual scholars (varying intercept model) and the number of data points for scholars are different. We then compare how well various sets of citation indicators 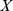 can estimate future citations 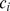 by comparing the explained variance 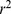 of the regression models with the same time horizons as defined by 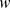 and 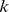.

To estimate future citations, we considered the effectiveness of 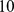 prominent citation indicators, namely the number of papers, the total number of citations, the career length, the average number of published papers per year, the average annual citations, the annual citations at the time of prediction, the average citations per paper, the *h* index, the *m* index, and the *g* index. The future citations of past and future papers were estimated with multi-level regression models. We compared for various time horizons, the coefficient of determination between models with different predictors (citation indicators).

For various 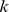
s and 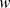
s , Table 1 compares how well the average citation per paper (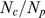), the 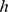 index and the annual received citations 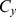 in the year of prediction 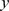, and also all the 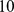 indicators can predict future citations.

**Table 1:** Explained variance of future citations estimated by the average number of citations per paper 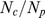 (1st column), the 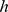 index (2nd column), the annual citations at the time of prediction 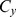 (3rd column), and all the 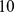 indicators (4th column).

Time windows	Predictors			
	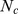 / 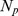	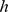 index	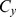	All 10 indicators
past, 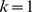	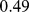	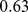	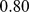	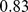
past, 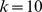	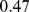	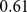	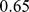	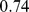
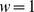 , 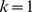	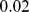	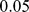	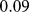	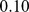
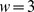 , 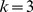	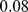	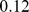	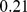	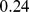
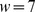 , 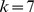	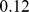	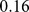	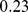	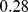
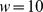 , 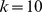	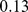	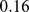	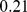	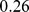

First, we consistently found that the annual citations 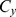 at the time of prediction 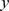 was the best predictor of future citations among the indicators (Table 1), and that including the remaining 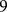 indicators increased the explained variance only by a small amount. The comparison between 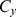 as a single predictor and all the 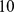 indicators (including 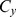) are illustrated in Fig. 4. The model parameter values for various 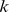
s and 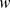
s with the single predictor 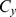 are shown in Table 2).

**Figure 4:**
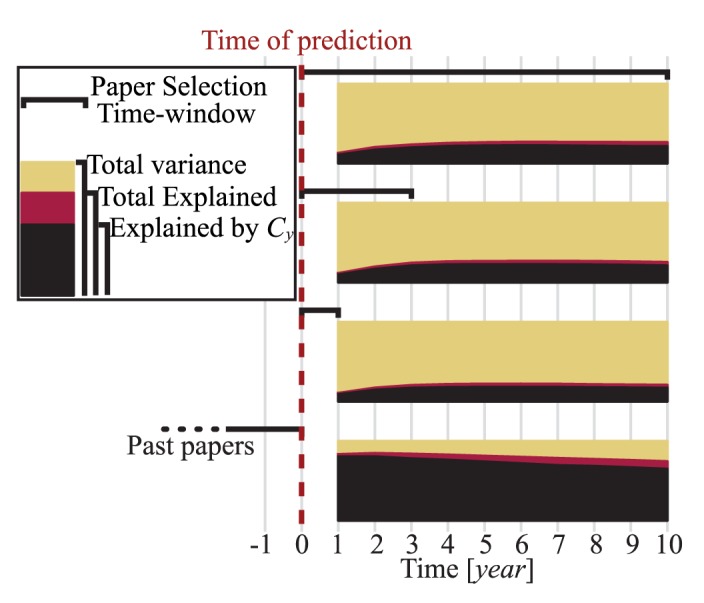
Explained variance of future citations. Future citations of published papers (bottom) and of future papers in 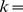 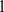, 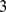, and 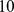 subsequent years (marked with paper selection time-windows in top 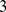) for 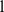 to 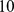 years after the time of prediction were estimated. Explained variance by annual citations (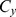) in black; Extra explained variance by including the remaining indicators in red.

**Table 2:** Future citations of published papers (Model 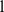 and 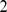) and future papers (Model 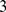, 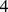, 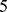 and 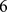) at the time of prediction as estimated by the annual citations at the time of prediction.

	Model 1	Model 2	Model 3	Model 4	Model 5	Model 6
Time windows	past, k = 1	past, k = 10	w = 1, k = 1	w = 3, k = 3	w = 7, k = 7	w = 10, k = 10
Intercept (SE)	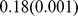	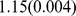	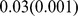	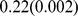	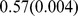	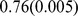
b (SE)	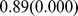	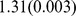	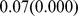	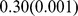	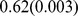	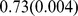
R-sq	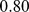	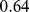	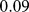	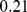	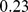	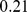
LR 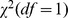	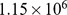	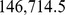	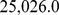	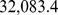	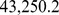	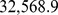
# observations	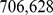	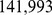	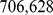	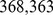	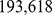	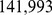
# scientists	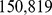	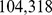	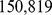	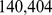	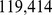	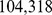

Second, for past papers, 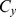 explained 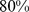 of the variance of future citations in the following year 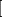
slope 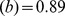, LR 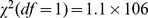, 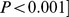. As its prediction power decayed over longer time horizons, 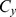 explained 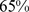 of the variance of future citations of past papers for a 10-year prediction (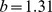, LR 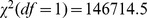, 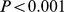). When we added the remaining 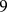 indices, the explained variance increased from 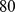 to 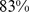 for the 1-year prediction, and from 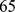 to 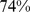 for the 10-year prediction. For short time horizons (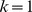), the future citations of past papers are much better estimated by 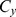, than the 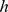 index or the average citation per paper (Table 1 for 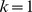 and 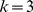).

Third, the explained variance of future citations to future papers were very small in all the considered models. For the longest prediction horizon (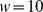, 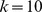), where the citations received in 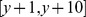 to papers published in the same period are estimated, not more than 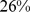 of variance was explained even when all the 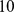 indicators were included (see last row of Table 1). A similarly weak (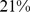 explained variance) estimation was achieved when 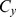 was the single estimator of our model. Estimating citations for shorter time horizons was generally harder. For the shortest prediction horizon 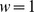, 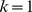 for example (third row in Table 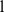), where the citations to papers published in year 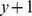 are estimated in the same year, only 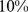 of variance is explained when all the 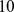 citation indicators were added in the model. Likewise, only 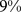 of variance was explained by 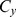. The other citation indicators perform even worse if used as single estimator of our regression model.

## Discussion

There is disagreement in the literature over the predictive power of the *h* index and that of the average number of citations per paper [1], [11]. In agreement with Hirsch's study [11], we found that the *h* index is a better predictor for the future citations of both published papers and future papers (Table 1). None of the studies, however, assessed 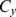, which we found to be the most powerful predictor of future citations. Discipline-wise analysis would require difficult choices in terms of classifying scholars and papers into disciplines. This classification requires extensive technical justifications, and we therefore reserve it for a future paper.

Our results have shown that the existing citation indices do not predict citations of future work well, and hence should not be given significant weight in evaluating academic potential. Including various indicators and testing various prediction time horizons, our results are still in agreement with Hirsch's study “past performance is not predictive of future performance.” [11]. Even combining multiple citation indicators did not significantly improve the prediction: apart from citation indicators, no better predictor of the impact of future work exists.
